# Experimental evaluation of sand fly collection and storage methods for the isolation and molecular detection of *Phlebotomus*-borne viruses

**DOI:** 10.1186/s13071-015-1192-8

**Published:** 2015-11-09

**Authors:** Maria Elena Remoli, Gioia Bongiorno, Claudia Fortuna, Antonella Marchi, Riccardo Bianchi, Cristina Khoury, Maria Grazia Ciufolini, Marina Gramiccia

**Affiliations:** Department of Infectious Parasitic and Immune-Mediated Diseases, Unit of Viral diseases and attenuated vaccine, Istituto Superiore di Sanità, Rome, Italy; Department of Infectious, Parasitic and Immune-Mediated Diseases, Unit of Vector-borne Diseases and International Health, Istituto Superiore di Sanità, Viale Regina Elena 299, 00161 Rome, Italy

**Keywords:** *Phlebotomus perniciosus*, *Phlebovirus*, Toscana virus, CDC light trap, Sticky trap, Viral isolation, RNA amplification

## Abstract

**Background:**

Several viruses have been recently isolated from Mediterranean phlebotomine sand flies; some are known to cause human disease while some are new to science. To monitor the *Phlebotomus*-borne viruses spreading, field studies are in progress using different sand fly collection and storage methods. Two main sampling techniques consist of CDC light traps, an attraction method allowing collection of live insects in which the virus is presumed to be fairly preserved, and sticky traps, an interception method suitable to collect dead specimens in high numbers, with a risk for virus viability or integrity. Sand flies storage requires a “deep cold chain” or specimen preservation in ethanol. In the present study the influence of sand fly collection and storage methods on viral isolation and RNA detection performances was evaluated experimentally.

**Methods:**

Specimens of laboratory-reared *Phlebotomus perniciosus* were artificially fed with blood containing Toscana virus (family *Bunyaviridae*, genus *Phlebovirus*). Various collection and storage conditions of blood-fed females were evaluated to mimic field procedures using single and pool samples. Isolation on VERO cell cultures, quantitative Real time-Retro-transcriptase (RT)-PCR and Nested-RT-PCR were performed according to techniques commonly used in surveillance studies.

**Results:**

Live engorged sand flies stored immediately at −80 °C were the most suitable sample for phlebovirus identification by both virus isolation and RNA detection. The viral isolation rate remained very high (26/28) for single dead engorged females frozen after 1 day, while it was moderate (10/30) for specimens collected by sticky traps maintained up to 3 days at room temperature and then stored frozen without ethanol. Opposed to viral isolation, molecular RNA detection kept very high on dead sand flies collected by sticky traps when left at room temperature up to 6 days post blood meal and then stored frozen in presence (88/95) or absence (87/88) of ethanol. Data were confirmed using sand fly pools.

**Conclusions:**

While the collection and storage methods investigated had not much impact on the ability to detect viral RNA by molecular methods, they affected the capacity to recover viable viruses. Consequently, sand fly collection and handling procedures should be established in advance depending on the goal of the surveillance studies.

## Background

Sand fly-borne viruses are distributed in large areas of the Old World (southern Europe, Africa, the Middle East, central and western Asia) with a wide spreading in the Mediterranean basin [1–3] where recent investigations have indicated that virus diversity is higher than initially suspected [3]. Therefore *Phlebotomus*-borne (*Ph*B) viruses have been recognized as an emerging health problem. Among sand fly-transmitted viruses, those belonging to the genus *Phlebovirus* (family *Bunyaviridae*) have great relevance for human health being the cause of meningitis, encephalitis and febrile illnesses. In particular, Sand fly Fever Sicilian Virus (SFSV) and Sand fly Fever Naples Virus (SFNV) are the causative agents of transient febrile illnesses in humans, while Toscana Virus (TOSV) exhibits peculiar neurotropism. TOSV infection is associated with aseptic meningitis or, less frequently, meningoencephalitis or encephalitis without meningitis. Asymptomatic or mild infections were reported in countries where it circulates [4–6].

Viruses belonging to the *Bunyaviridae* family have a genome composed of three negative-sense RNA segments designated S, M, and L that encode the nucleocapsid (N), envelope glycoproteins (Gn and Gc), and RNA-dependent RNA polymerase (L), respectively [7]. Their genomic organization makes possible genetic molecular evolution by antigenic drift, antigenic shift (genetic reassortment), and genetic recombination [8, 9]. These genomic properties, allowing the appearance of new variants, make them good candidates as emerging human pathogens. Indeed, in the last five years, new phleboviruses have been identified suggesting that many of them still remain to be discovered [1–3, 10, 11]. Therefore active surveillance in vectors represents an important tool to control the viral spreading and circulation. Accurate and timely detection of viruses potentially transmitted by sand flies is an essential component of surveillance and control programs. Surveillance relies on the identification of viruses from field-collected sand flies through detection of viral RNA and/or live viral particles. Because of the viral fragility, efforts should be made to handle and process the sand fly specimens so as to minimize exposure to conditions that could degrade the virus. Ideally, a “deep cold chain” should be maintained from field collection of specimens until laboratory processing.

Adult sand flies of both sexes can be collected by several methods, either when foraging at night or resting during the day. A variety of sampling methods are available that can be divided into two groups, one of which consists of techniques for catching living flies while the other is suitable only for dead specimens [12, 13]. Once the ecology and habits of the sand fly population are known, one or two sampling approaches can be chosen. The most common procedures consists of light traps, such as battery-operated CDC light traps, to catch host-seeking females, and adhesive sticky traps to catch resting flies. Variation in climatic conditions such as temperature, humidity and wind speed can affect sampling success. CDC light traps are expensive and not very practical; they are used extensively in field studies because they are less labor intensive and positioning poses fewer problems of standardization. They allow the collection of live insects in which the virus is presumed to be fairly preserved. In contrast, sticky traps, an interception method mainly used to determine the relative density and seasonal trend of phlebotomine populations, are less expensive and practical enough. They consist of paper sheets impregnated with castor oil inserted in places where sand flies are resting. These traps are generally inexpensive and easy to manufacture in large numbers and stored until required. However, this method provides only dead specimens, although in high numbers and from different environments.

Preservation techniques for storage and transportation of sand flies depend on the purpose for which the specimens were collected. If possible, it is best to transport adult specimens to the laboratory alive. For taxonomic studies specimens can be preserved dry or in 70 % ethanol (EtOH). However, most studies involving molecular-based protocols can use dried, fresh, frozen or alcohol-preserved specimens. For preservation of nucleic acids, 95–100 % EtOH can be used without immediate need for refrigeration [14–16]. The freezing storage of the insects increases the period for management of the specimens.

The possibility to detect viral RNA in specimens after several days from their death and under unsuitable environmental conditions remains to be ascertained. Although several studies in this field were reported on the arbovirus detection in mosquitoes [17–19], little is known on the sand flies and *Ph*B-viruses. In the present study an accurate analysis of the influence of sand flies collection and subsequent storage methods on the virus isolation and viral RNA detection was assessed. For this purpose the experimental infections using laboratory-reared *Phlebotomus perniciosus* artificially infected with TOSV, were performed to: a) evaluate the ability to detect virus and viral RNA in alive or dead infected sand flies by isolation and RNA detection; b) to assess the influence of EtOH presence in sand flies storage for viral detection; c) to evaluate the RNA detection by a specific quantitative real time (q) Retro-transcriptase (RT)-PCR and a *Phlebovirus* Nested-RT-PCR commonly used in viral entomological surveys.

## Methods

### Ethics statement

This study was carried out in accordance with the recommendations of the Animal Experimentation protocol. At the time when experiments were performed, the use of laboratory animals in Italy was regulated by legislative Decree no. 116/92, which implemented the European Directive 86/609/EEC on laboratory animal protection. In accordance with this legislation the presence and approval of an Ethical Committee is not required; however local welfare veterinarians had the same functions as IACUCs. In particular, at Istituto Superiore di Sanità, the veterinarians working for the Service for Biotechnology and Animal Welfare performed the functions of local IACUC; they approved animal research protocols and they verified that the guidelines of legislative Decree no. 116/92 on animal welfare were strictly and constantly implemented.

### Experimental protocol

#### Virus

TOSV, ISS PHL 32 strain, isolated from *P. perniciosus* sand flies collected in Sesto (Florence, Tuscany, Italy) in 1981 [20], was used for the experimental infections. The strain was propagated on VERO cells, stored at - 80 °C in aliquots and then titred by Plaque Forming Units/mL (PFU/mL) (4.86 x 10^7^ PFU/mL).

#### P. perniciosus *laboratory colony*

Laboratory-reared *P. perniciosus* sand flies (origin: Madrid, Spain), received in ISS since 2010, were used; rearing conditions were 28°–29 °C for larval stages and 27 °C for adults, with photoperiod of 15/9 h light/dark and 90 % RH [21, 22]. The *P. perniciosus* colony resulted virus free by previous laboratory analysis.

#### P. perniciosus *experimental infection*

Infection experiments were performed in BSL2 cabinet at room temperature of 28–29 °C and about 90 % RH. The infectious blood meal was composed of four parts of mechanically defibrinated rabbit blood and one part of viral seed, with a final concentration of 9.72 x 10^6^ log_10_ PFU/mL. The viral titre of ISS PHL 32 seed, used in these experiments, was in accordance with previous studies and considered to be adequate to obtain 100 % of infected sand flies after the blood meal [23, 24]. Three to 7 day-old sand fly females, together with males, were allowed to feed for 2–3 h through a chicken skin membrane covering the base of a glass feeder containing the blood-virus mixture, maintained at 36.5–37.5 °C by a warm water circulation system. Two experimental infections were performed to ensure the reproducibility of our system, which aimed at the analysis of single blood-fed individuals. After the second experimental infection, blood-fed specimens were also tested in pools of 20 insects - a usual sample in field surveys - among which only one was blood-fed and 19 were naïve adults from colony cages not involved in the infection experiments.

#### Collection, preparation and storage of blood-fed sand flies

**Fig. 1:**
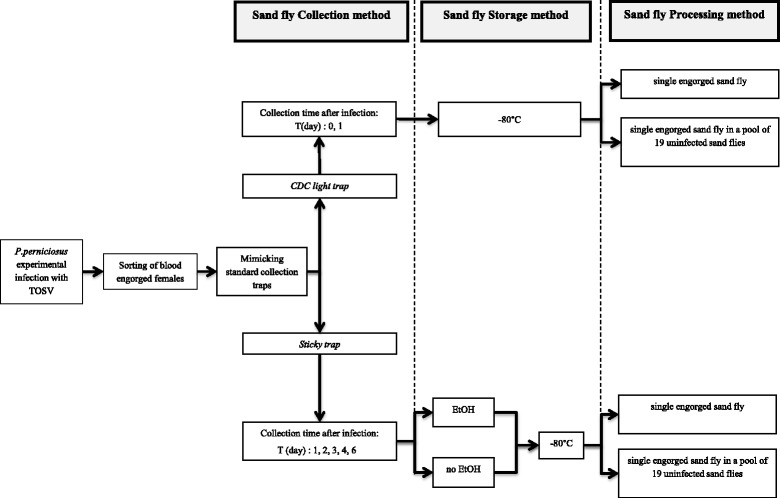
Schematic algorithm of the *Phlebotomus perniciosus*-Toscana virus experimental design. Diagram representing the experimental plan to investigate the effect of sand fly collection and storage methods on virus viability and RNA detection

A diagram of experimental procedures is shown in Fig. 1. Immediately after the infectious blood meal, engorged females were sorted, killed by carbon dioxide and then handled to mimic the two main sand fly collection methods. A group of specimens were immediately frozen at −80 °C, to reproduce the ideal situation of alive sand flies (AS) caught by CDC light trap (*AS-CDC*) followed by maintenance through a “deep cold chain”. An additional group of dead insects (DS) was left at room temperature for 24 h and then stored at −80 °C, to mimic the recovery of dead sand flies frequently found on the bottom of a CDC light trap after one-day collection (*DS-CDC*). A large group of engorged females was carefully put onto sticky traps (St, consisting of 20 x 20 cm castor oil impregnated papers) which were left at room temperature. Every day a subgroup of these specimens was removed using a EtOH-soaked fine small brush, for up to 6 days post blood meal (p.b.m.). To evaluate the effect of EtOH storage, 50 % of these DS specimens were individually preserved in EtOH 99 % (*DS-St + EtOH*) and then stored at −80 °C. To avoid exposure to EtOH with possible degrading effects on viral particles, the remaining DS specimens were stored dry at −80 °C after mild washing in PBS pH 7.4 (to remove EtOH contamination by the soaked brush) and drying on filter paper for 5 min (*DS-St*).

### Sand fly sample processing

Blood-fed sand flies were assayed for TOSV presence both individually and within pools. Specimens were homogenized and suspended in 1 mL of Hank’s solution containing 7.5 % bovine albumin and 1 % antibiotic-antimycotic mix (Invitrogen, Gibco) [24]. Before being processed, *DS-St + EtOH* insects were removed from EtOH and left to dry under BSL-2 cabinet for 10 min at room temperature. The samples were then analyzed by: i) TOSV isolation in VERO cell cultures; ii) TOSV RNAs detection by specific quantitative real time qRT-PCR and iii) Phleboviruses RNA detection by Nested-RT-PCR using degenerated primers for SFNV complex.

#### Virus isolation

Viral isolation was carried out as described by Verani et al. [25]. Briefly, after centrifugation of the homogenate at 3,000 × g for 30 min, 100 μL of the supernatant fluid was seeded on a confluent VERO cells monolayer. After 1 h incubation at 37 °C, 2 mL of medium, consisting of Dulbecco’s MEM, 2 % FBS, 1 % antibiotic-antimycotic mix (Invitrogen, Gibco), was added. VERO cell cultures were examined daily for 14 days for cytopathic effect (CPE). The viral isolation success was expressed in function of CPEs obtained for each sand fly groups and subgroups as: very high (90–100 %), high (60–80 %), moderate (30–50 %), low (10–20 %) and nil (0 %).

#### RNA virus detection

- Quantification of TOSV RNAs by qRT-PCR
  RNA was extracted from the homogenized sand flies using the QIAamp viral RNA kit (Qiagen Inc., Valencia, CA, USA) and stored at −80 °C. The qRT-PCR was performed by using TOSV TaqMan primers and probe amplifying 89 bp of the N gene [26]. Briefly, 7 μL of RNA was combined with 20 pmol of each primer and 4 pmol of the FAM- and TAMRA-labelled probe in a 20 μL total reaction volume by using the RNA Virus Master Roche (Roche Diagnostics, Basel, CH). The RNAs were amplified in a CFX96 Touch™ Real-Time PCR Detection (Bio-Rad), with the following cycling times and temperatures: 1 cycle of retro transcription at 50 °C for 30 min, 1 cycle at 94 °C for 2 min and 45 cycles of 94 °C for 15 s, 60 °C for 30 s (acquisition mode: single), and 72 °C for 2 s (Ramp Rate: 2 °C/s). TOSV RNA quantification was determined by comparing crossing points values to standard curve based on data acquired from 10-fold serial dilutions of virus stocks with estimated concentration by titration on VERO cells and expressed as Log_10_ PFUeq/mL.
- Detection of viral RNA by Nested-RT-PCR
  In order to mimic natural field conditions to detect different phleboviruses, viral RNA was amplified by Nested-RT-PCR using conventional degenerated consensus primers specific for SFNV complex of *Phlebovirus* (including TOSV) and targeting the nucleoprotein (N) gene in the S RNA segment [27]. The Nested-RT-PCR was performed using Super script One step RT-PCR System Kit (Invitrogen, Gaithersburg, MD) and PCR SuperMix (Invitrogen), according to the manufacturer’s recommendations. The PCR conditions were those previously described [27]. PCR products were analyzed in a 2 % TAE agarose electrophoresis gel.

#### Statistical analysis

Significance for proportions was tested by Fisher’s exact test (significance level of *p* <0.05). qRT-PCR molecular data collected at each time point were considered and means of viral titres from each sand fly groups and subgroups were compared for significance using the non-parametric test of Mann–Whitney U test (*p* <0.05). In addition the performance of the distribution respect to the variable average time across different sand fly groups and subgroups was evaluated using the non-parametric trend test developed by Cuzick [28] (*nptrend* <0.05). Statistical analyses were performed using GraphPad Prism Software, version 5.00 for Windows (San Diego, California, USA).

## Results

A total of 1674 *P. perniciosus* females were employed in two artificial feeding experiments, resulting in 658 blood-fed females (average feeding efficiency: 39.3 %; range: 25.4–45.5 %). Two hundred and eight engorged females were processed and analyzed. Ten to fifteen individual insects were tested for *AS*- and *DS-CDC* groups, while 5 specimens were analyzed for *DS-St* and *DS-St + EtOH* daily subgroups. Each groups and subgroups of the two experimental infections were not statistically different (Table 2).

### Individual sand fly processing

#### Effect of collection and storage methods on virus viability

As shown in Table 1, all 20 *AS-CDC* specimens showed 100 % TOSV isolation rate, confirming the presence of viable virus in all living insects; the viability of TOSV remained very high (26/28; 93 %) in 28 *DS-CDC* specimens with 13/13 specimens in the 1^st^ experiment (100 %) and 13/15 (87 %) in the 2^nd^ one. By contrast, only a moderate isolation rate was obtained in 10/30 (33 %) *DS-St* insects until 3 days p.b.m. (*p* = 0.0001 at the Fisher’s exact text), which progressively decreased to low (10 %) in 1/10 (days 4 p.b.m.) to nil rate (0 %) in 10 *DS-St* insects by days 6 p.b.m. Finally, all 50 *DS-St + EtOH* specimens showed no CPE at any time, proving evidence of viral isolation failure at these conditions.

**Table 1:** TOSV isolation by CPE in VERO cell cultures from sand flies individually processed

Sandflies collection and storage methods	Day post infection	1^st^ Experimental infection	2^nd^ Experimental infection	Total
			Pos/tested (%)	
*AS-CDC*	0	10/10 (100)	10/10 (100)	20/20 (100)
*DS-CDC*	1	13/13 (100)	13/15 (87)	26/28 (93)
*DS-St*	1	2/5 (40)	2/5 (40)	4/10 (40)
	2	2/5 (40)	1/5 (20)	3/10 (30)
	3	2/5 (40)	1/5 (20)	3/10 (30)
	4	1/5 (20)	0/5 (0)	1/10 (10)
	6	0/5 (0)	0/5 (0)	0/10 (0)
*DS-St + EtOH*	1	0/5 (0)	0/5 (0)	0/10 (0)
	2	0/5 (0)	0/5 (0)	0/10 (0)
	3	0/5 (0)	0/5 (0)	0/10 (0)
	4	0/5 (0)	0/5 (0)	0/10 (0)
	6	0/5 (0)	0/5 (0)	0/10 (0)

*AS-CDC* alive blood-fed sand flies stored at −80 °C immediately after TOSV infection, *DS-CDC* dead blood-fed sand flies recovered after 1 day post infectious blood meal at room temperature, *DS-St* dead blood-fed females left at room temperature on sticky papers and stored without EtOH, *DS-St + EtOH* dead blood-fed sand flies left at room temperature on sticky papers and then stored with EtOH, CPE cytopathic effect

#### Effect of collection and storage methods on TOSV RNA yield by qRT-PCR

Viral RNAs recovered from all *AS-*and *DS-CDC* specimens were successfully amplified and showed high TOSV mean titres (Table 2). All RNAs recovered from *DS-St* specimens were also amplified, however viral RNA titres were significant lower than in *AS-* (*p* = 0.001) and *DS-CDC* samples (*p* = 0.002 and *p* = 0.008 for the two experiments respectively), remaining approximately constant in all time-points (*nptrend* = 0.371 and *nptrend* = 0.317 for both experiments respectively). The comparison of TOSV RNA amplification and mean viral titres at the same time-points between *DS-St* and *DS-St + EtOH* insects, suggests that they were influenced in some way by the EtOH presence. In the 1^st^ experiment, not all the *DS-St + EtOH* insects collected after 1 or 2 days p.b.m. were found positive; furthermore, in both experiments RNA TOSV titres in *DS-St + EtOH* samples were consistently and significantly lower than in *DS-St* samples (*p* = 0.0033 and *p* = 0.0001 for both experiments respectively).

**Table 2:** TOSV RNA quantification by qRT-PCR in sand flies individually processed

Sandflies collection and storage methods	Day post infection	1^st^ Experimental infection	2^nd^ Experimental infection	U Mann Whitney Test	Total
		Pos/tested (%) mean ± SD		*P* value	Pos/tested (%)
*AS-CDC*	0	10/10(100) 3.74 ± 0.34	10/10(100) 3.61 ± 0.54	0.393	20/20 (100)
*DS-CDC*	1	13/13(100) 3.78 ± 0.71	15/15(100) 3.46 ± 0.60	0.413	28/28 (100)
*DS-St*	1	5/5(100) 3.07 ± 0.27	5/5(100) 2.60 ± 0.47		
	2	5/5(100) 2.86 ± 1.27	5/5(100) 3.11 ± 0.20		
	3	5/5(100) 3.10 ± 0.54	5/5(100) 2.54 ± 0.33	0.548	50/50 (100)
	4	5/5(100) 3.16 ± 0.26	5/5(100) 2.95 ± 0.29		
	6	5/5(100) 3.11 ± 0.70	5/5(100) 3.23 ± 0.10		
*DS-St + EtOH*	1	4/5(80) 2.63 ± 0.77	5/5(100) 1.47 ± 0.38		
	2	3/5(60) 2.06 ± 0.02	5/5(100) 1.47 ± 0.64		
	3	5/5(100) 2.29 ± 0.51	5/5(100) 2.33 ± 0.48	0.310	47/50 (94)
	4	5/5(100) 2.84 ± 0.76	5/5(100) 2.63 ± 0.69		
	6	5/5(100) 3.01 ± 0.44	5/5(100) 2.65 ± 0.56		
Total		73	75		145/148 (98)

*SD* standard deviation, *AS-CDC* alive blood-fed sand flies stored at −80 °C immediately after TOSV infection, *DS-CDC* dead blood-fed sand flies recovered after 1 day post infectious blood meal at room temperature, *DS-St* dead blood-fed females left at room temperature on sticky papers and stored without EtOH, *DS-St + EtOH* dead sand flies left at room temperature on sticky papers and then stored with EtOH

#### Effect of collection and storage methods on viral RNA detection by Nested-RT-PCR

As shown in Table 3, the RNAs obtained from all 6 *AS-* and 6 *DS-CDC* sand flies were amplified by Nested-RT-PCR for SFNV complex of *Phlebovirus*. In 38 *DS-St* insects the viral RNA was detected in almost all of sand flies tested (37/38, 97.4 %), except in the 1^st^ experiment where, on day 2 p.b.m., 1/3 specimens resulted negative. This is in accordance with the high diversity in RNA titres of these samples observed at the TOSV qRT-PCR analysis (SD = 1.27) (Table 2). Finally, the 45 *DS-St + EtOH* samples showed a positivity rate by Nested-RT-PCR (41/45, 91.1 %) similar to that obtained by TOSV qRT-PCR assays (47/50, 94.0 %)(*p* = 0.7 at the Fisher’s exact text): all infected sand flies were positive with the exception of the samples collected at day 1 and 2 p.b.m. (Tables 2 and 3).

**Table 3:** TOSV RNA detection by Nested-RT-PCR for SFNV complex of *Phlebovirus* in sand flies individually processed

Sand flies collection and storage methods	Day post infection	1^st^ Experimental infection	2^nd^ Experimental infection	Total
			Pos/tested (%)	
*AS-CDC*	0	3/3 (100)	3/3 (100)	6/6 (100)
*DS-CDC*	1	3/3 (100)	3/3 (100)	6/6 (100)
*DS-St*	1	5/5 (100)	4/4 (100)	9/9 (100)
	2	2/3 (67)	4/4 (100)	6/7 (86)
	3	4/4 (100)	4/4 (100)	8/8 (100)
	4	3/3 (100)	4/4 (100)	7/7 (100)
	6	3/3 (100)	4/4 (100)	7/7 (100)
*DS-St + EtOH*	1	4/5 (80)	3/4 (75)	7/9 (78)
	2	3/5 (60)	4/4 (100)	7/9 (78)
	3	5/5 (100)	4/4 (100)	9/9 (100)
	4	5/5 (100)	4/4 (100)	9/9 (100)
	6	5/5 (100)	4/4 (100)	9/9 (100)

*AS-CDC* alive blood-fed sand flies stored at −80 °C immediately after TOSV infection, *DS-CDC* dead blood-fed sand flies recovered after 1 day post infectious blood meal at room temperature, *DS-St* dead blood-fed females left at room temperature on sticky papers and stored without EtOH, *DS-St + EtOH* dead blood-fed sand flies left at room temperature on sticky papers and then stored with EtOH

**Fig. 2:**
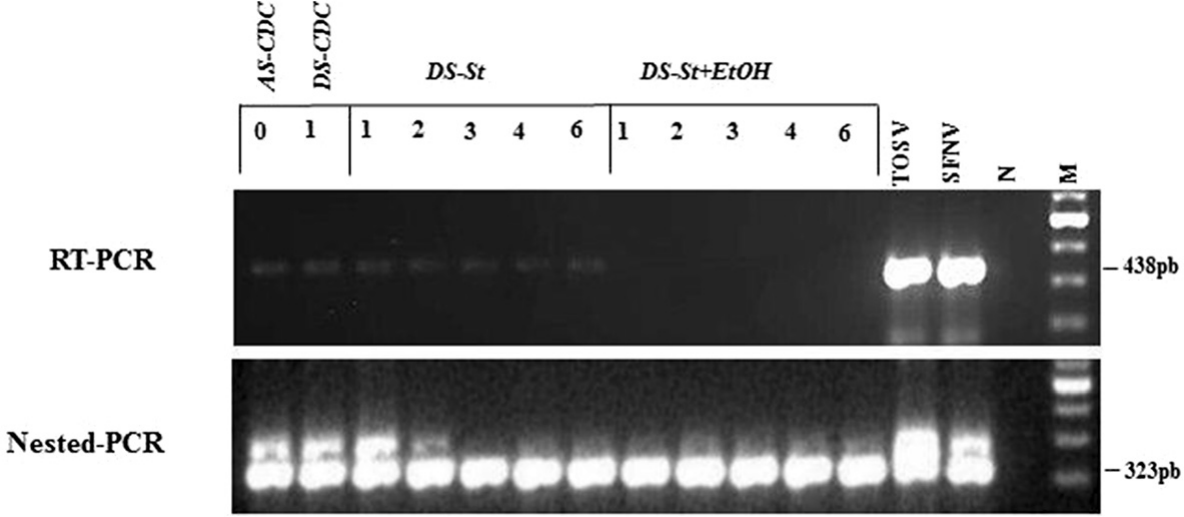
Gel electrophoresis of RT- and Nested-RT-PCR amplicons of a portion of SFNV complex of *Phlebovirus* N gene. *AS-CDC*: alive blood-fed sand flies stored at −80 °C immediately after TOSV infection; *DS-CDC*: dead blood-fed sand flies recovered after 1 day post infectious blood meal at room temperature; *DS-St*: dead blood-fed females left at room temperature on sticky papers and stored without EtOH; *DS-St + EtOH*: dead blood-fed sand flies left at room temperature on sticky papers and then stored with EtOH; TOSV: Toscana Virus; SFNV: Sand fly Fever Naples Virus; N: negative control; M: 100 bp ladder

An example of RT-PCR and Nested-RT-PCR gel electrophoresis is presented in Fig. 2. TOSV RNA positivity by RT-PCR is shown for all the *AS-CDC*, *DS-CDC* and *DS-St* specimens, whereas no bands are observed for *DS-St + EtOH* sand flies, while all of them were found positive by Nested-RT-PCR.

### Sand flies processed as pools

Considering that phlebotomine specimens are usually analyzed as pools of ~20 insects in viral surveillance studies, we investigated this aspect in the 2^nd^ experiment by testing 5 pools for each groups and subgroups. As shown in Table 4, viable virus was recovered in VERO cell cultures from all 5 *AS-* and 5 *DS-CDC* pools tested. Viral isolation was successful in 4/5 (80 %) of *DS-St* pools recovered on day 1 p.b.m., however this rate decreased significantly, with fluctuations ranging from 40 % (2/5 at day 2 and 4) to 20 %, (1/5 at day 3) for pools collected from days 2 up to days 4 p.b.m.. The lack of viral growth from 5 *DS-St* pools on day 6 p.b.m. correlated well with results from similar specimens processed individually. CPE was not obtained from all 25 *DS-St + EtOH* pools examined at any time-point. TOSV qRT-PCR results were consistent with mean viral titres observed in specimens processed individually (Tables 2 and 4) and the differences were not significant (*p* = 0.799). TOSV RNA titres were achieved from *DS-St* and *DS-St + EtOH* pools at all days of collection remaining approximately constant (*nptrend* = 0.549 for *DS-St*, *nptrend* = 0.317 for *DS-St + EtOH*). As observed in sand flies processed individually, on day 1 and 2 p.b.m a significantly lower RNA level was detected in *DS-St + EtOH* pools as compared with *DS-St* pools (*p* = 0.0001) suggesting a negative EtOH viral influence. Finally, Nested-RT-PCR for SFNV complex of *Phlebovirus* analysis showed positive amplification with all 43 sand fly pools tested (Table 4) in accordance with results from individually processed specimens (Table 3).

**Table 4:** TOSV isolation and RNA detection in sand fly processed as pools

Sandflies collection and storage methods	Day post infection	TOSV isolation	RNA detection	
			TOSV RTqPCR	Nested-RT-PCR for SFNV complex of *Phlebovirus*
		Pos/tested (%)	Pos/tested (%) mean ± SD	Pos/tested (%)
*AS-CDC*	0	5/5 (100)	5/5 (100) 3.17 ± 0.22	3/3 (100)
*DS-CDC*	1	5/5 (100)	5/5 (100) 2.95 ± 0.28	3/3 (100)
*DS-St*	1	4/5 (80)	5/5 (100) 3.04 ± 0.18	4/4 (100)
	2	2/5 (40)	5/5 (100) 2.97 ± 0.39	4/4 (100)
	3	1/5 (20)	5/5 (100) 3.14 ± 0.28	3/3 (100)
	4	2/5 (40)	5/5 (100) 2.95 ± 0.17	3/3 (100)
	6	0/5 (0)	5/5 (100) 3.19 ± 0.15	3/3 (100)
*DS-St + EtOH*	1	0/5 (0)	5/5 (100) 1.30 ± 0.28	4/4 (100)
	2	0/5 (0)	5/5 (100) 2.20 ± 0.20	4/4 (100)
	3	0/5 (0)	5/5 (100) 3.03 ± 0.63	4/4 (100)
	4	0/5 (0)	5/5 (100) 1.94 ± 0.37	4/4 (100)
	6	0/5 (0)	5/5 (100) 2.77 ± 0.38	4/4 (100)

*SD* standard deviation; *AS-CDC* alive blood-fed sand flies stored at −80 °C immediately after TOSV infection; *DS-CDC* dead blood-fed sand flies recovered after 1 day post infectious blood meal at room temperature; *DS-St* dead blood-fed females left at room temperature on sticky papers and stored without EtOH; *DS-St + EtOH* dead blood-fed sand flies left at room temperature on sticky papers and then stored with EtOH

## Discussion

Active surveillance is considered the most important approach for providing early warning and predictive capacity in viral epidemics. Several field-based studies combining entomological and virological aspects are in progress to monitor the distribution of *Ph*B-viruses and their vectors. Recent investigations have indicated that the *Phlebovirus* diversity in the Mediterranean basin is higher than initially suspected and novel viruses are reported every year [10, 11, 29–34]. It would be necessary to improve the overall methodology in order to implement timely, accurate and meaningful vector surveys and virus detection for estimating the transmission risk in endemic areas and drawing detailed maps of *Ph*B-virus disease occurrence. Therefore the standardization of reliable and affordable sand fly collection and storage methods is important to harmonize surveillance activities. The genomic and structural nature of phleboviruses makes them highly fragile and efforts should be made to handle and process sand flies so as to avoid virus inactivation and RNA degradation. Alive specimens associated to a “deep cold chain” have always been considered the best method for both viral isolation and RNA identification, underlying that specimens collected by sticky traps should not be used for virus isolation studies because the impregnation oil interferes with cell culture [35]. Several studies on mosquitoes reported that lack of a cold chain does not appear to reduce the ability to detect arboviral RNA after the insect death [17, 18, 36–40]. In these studies the authors highlighted that the alive or frozen mosquitoes were not a prerequisite for viral RNA amplification although viral isolation results were significantly affected [17, 18, 37, 40]. Although, up to now, no standardized methods are reported in literature it is established practice among researchers involved in arboviruses investigations, to collect sand flies by CDC traps and to store them immediately at −80 °C in order to obtain a successful Phleboviruses detection from field-collected sand flies [10, 11, 29–31, 41]. However, up to now, no studies have reported on the effects of sand flies collection and storage methods on *Ph*B-viruses detection. The present study firstly provides basic conclusions for *Ph*B-viruses guidelines to a correct practice between entomologists and virologists.

As expected and previously suspected, virus isolation on VERO cell cultures was successful for all the alive females stored immediately at −80 °C after having a TOSV infectious-blood meal (*AS-CDC*). The percentage of positive isolations from dead females left at room temperature (*DS-CDC*) remained very high (>90 %) after 1 day p.b.m. Viral isolation rate was moderate (>30 %) for *DS-St* sand flies processed from day 1 through to day 3 p.b.m., but it was drastically reduced from low (10 %) to nil thereafter in both sand flies processed individually or in pools. Our results proved that storage in EtOH, a common insect fixative widely used in entomological taxonomy and DNA analysis [13, 15, 16, 42], is not generally suitable for TOSV culture isolation from sand flies. This is probably due to the biophysical properties of enveloped RNA-viruses, including TOSV, which generally exhibit low resistance to the environmental conditions. On the other hand, our results suggest the possibility to still isolate *Ph*B-viruses after a few days from sand fly death using careful washing of specimens by PBS pH 7.4 to eliminate EtOH contamination (*DS-St*) with a moderate (>30 %) or low (10 %) isolation rate up to 4 p.b.m. (Table 1).

In accordance with results obtained with mosquitoes [17, 18, 37, 40], our studies demonstrated that TOSV RNA persisted up to 6 days p.b.m. in the majority of the specimens stored with or without EtOH, and tested by TOSV qRT-PCR and Nested-RT-PCR for SFNV complex of *Phlebovirus* (Tables 2 and 3). Of note, regarding *DS-St + EtOH* sand flies we observed that, in the 1^st^ experiment, some specimens recovered at days 1 and 2 p.b.m. were negative by both RNA amplification methods (Tables 2 and 3). On the same days in the 2^nd^ experiment, specimens processed both individually and in pools showed the lowest TOSV qRT-PCR titres (Tables 2 and 4). It could be hypothesized that castor oil, the impregnating oil of sticky traps, was still wet during the first 2 days and, as with EtOH, it could interfere with RNA extraction and affect RNA yields.

The present study demonstrated that using fast RT-PCR procedures (e.g. qRT-PCR and Nested-RT-PCR), the detection of viral RNA was possible in live sand flies as well as in dead specimens left on a sticky trap up to six days and then stored frozen with or without EtOH (*DS-St* and *DS-St-EtOH*). Therefore sand flies preserved in EtOH, a storage method widely used in sand fly taxonomy, can also be used for molecular studies allowing a viral genome RNA identification. However some interference on RNA detection was shown, so that lower viral loads and rates of infected individuals are to be expected in natural field conditions [16]. Finally viral isolation was quite successful in all alive specimens associated to a deep cold chain, and in *DS-CDC* and *DS-St* specimens recently collected.

## Conclusion

The present study confirms that the *Ph*B-viruses detection from sand flies is influenced by the collection and storage methods of these insects. Sand flies collected alive by CDC light traps are suitable for successful viral isolation and RNA identification. The use of sticky traps is more suitable for genome viral identification than for viral isolation. It should be underlined that qRT-PCR or Nested-RT-PCR for SFNV complex of *Phlebovirus*, using specific and/or degenerated primers, can only provide information on viral genomes for which diagnostic primers were designed. Therefore viral isolation is a prerequisite for biological and phylogenetic analysis of novel *Ph*B-viruses not yet characterized.

Consequently, sand fly collection and handling procedures should be established in advance depending on the goal of the surveillance studies. A coordination among virologists and entomologists should be strongly encouraged before any epidemiological or surveillance surveys are planned.

## Abbreviations

AS-CDC: alive engorged sand flies stored at −80 °C immediately after TOSV infection
CPE: cytopathic effect
DS: dead sand flies
*DS-CDC*: dead blood-fed sand flies left at room temperature and stored after 1 day post blood meal
*DS-St*: dead blood-fed sand flies left at room temperature on sticky papers and stored without EtOH
*DS-St + EtOH*: dead blood-fed sand flies left at room temperature on sticky papers and then stored with EtOH
EtOH: ethanol
*P. perniciosus*: *Phlebotomus perniciosus*
p.b.m: post blood meal
PFU/mL: Plaque Forming Units/mL
*Ph*B: *Phlebotomus*-borne
qRT-PCR: quantitative real time (q) Reverse Transcriptase (RT)-PCR
SFNV: Sand fly Fever Naples Virus
SFSV: Sand fly Fever Sicilian Virus
St: Sticky traps
TOSV: Toscana Virus
